# Supplementary material for: GABA Application Enhances Drought Stress Tolerance in Wheat Seedlings (Triticum aestivum L.)
Source: Plants (Basel). 2023 Jun 29;12(13):2495. doi: 10.3390/plants12132495 (PMC10346274; doi:10.3390/plants12132495)
Supplement: Supplementary file 1 [file plants-12-02495-s001.zip › plants-2404012-supplementary.pdf]

Supplementary material

GABA application enhances drought stress tolerance in wheat seedlings (*Triticum aestivum* L.)

Table S1 Pearson correlations for variables of phenolics contents and enzymes of phenolics biosynthesis in wheat seedlings with GABA treatment

Indicators	Total phenolic	Total phenolic acid	*p*-hydroxybenzoic acid	vanillic acid	syringic acid	*p*-coumaric acid	ferulic acid	sinapic acid	PAL	C4H
Total phenolic acid	0.953**									
p-hydroxybenzoic acid	0.504	0.492								
vanillic acid	0.468	0.522*	0.750**							
syringic acid	0.901**	0.895**	0.145	0.412						
p-coumaric acid	0.681**	0.753**	0.703**	0.878**	0.596*					
ferulic acid	0.941**	0.975**	0.370	0.340	0.880**	0.595*				
sinapic acid	0.866**	0.937**	0.399	0.532*	0.904**	0.709**	0.895**			
PAL	0.075	-0.15	-0.104	-0.337	-0.183	-0.307	0.118	-0.256		
C4H	0.927**	0.923**	0.503	0.378	0.869**	0.592*	0.938**	0.802**	0.232	
4CL	0.444	0.360	0.103	-0.078	0.243	-0.020	0.472	0.140	0.859**	0.586

Note: * represent significant level at $p < 0.05$. ** represent significant level at $p < 0.01$.

Table S2 Pearson correlations for variables of phenolics contents and antioxidant capacity in wheat seedlings with GABA treatment

Indicators	Total phenolic	Total phenolic acid	*p*-hydroxybenzoic acid	vanillic acid	syringic acid	*p*-coumaric acid	ferulic acid	sinapic acid	ABTS values
Total phenolic acid	0.953**								
p-hydroxybenzoic acid	0.504	0.492							
vanillic acid	0.468	0.522*	0.750**						
syringic acid	0.901**	0.895**	0.145	0.412					
p-coumaric acid	0.681**	0.753**	0.703**	0.878**	0.596*				
ferulic acid	0.941**	0.975**	0.370	0.340	0.880**	0.595*			
sinapic acid	0.866**	0.937**	0.399	0.532*	0.904**	0.709**	0.895**		
ABTS values	0.952**	0.924**	0.486	0.408	0.843**	0.590*	0.941**	0.793**	
DPPH values	0.959**	0.976**	0.458	0.445	0.955**	0.673**	0.968**	0.922**	0.939**

Note: * represent significant level at $p < 0.05$. ** represent significant level at $p < 0.01$.
